# Arm movement strategies did not influence emotional state and static postural control during height-induced postural threat in children and young adults

**DOI:** 10.3389/fnhum.2025.1635330

**Published:** 2025-08-06

**Authors:** Anna M. Wissmann, Mathew W. Hill, Thomas Muehlbauer, Johanna Lambrich

**Affiliations:** ^1^Division of Movement and Training Sciences/Biomechanics of Sport, University of Duisburg-Essen, Essen, Germany; ^2^Center for Physical Activity, Sport and Exercise Sciences, Coventry University, Coventry, United Kingdom

**Keywords:** postural control, anxiety, perception, upper body strategy, age

## Abstract

**Background:**

Empirical evidence in adults suggests that height-induced postural threat led to an increased reliance on an ankle control strategy (i.e., postural “stiffening” response). However, little is known whether children (i.e., due to ongoing maturation) show a similar pattern and how this is affected by the used arm movement strategy.

**Objective:**

The objective of this study was to compare the effects of different arm movement strategies on subjective and objective indicators related to standing at or above ground-level in children versus young adults.

**Methods:**

Twenty-six children (age: 9.8 ± 0.6 years) and 23 young adults (age: 24.7 ± 4.0 years) performed the tandem stance whilst standing at both ground-level (no threat) and 80 cm above ground (threat). During both, participants performed the task with free and restricted arm movements. Self-reported emotional state outcomes (i.e., balance confidence, fear of falling, perceived instability, conscious balance processing) were assessed and used as subjective indicators. Static balance outcomes (i.e., postural sway amplitude, frequency, and velocity) were measured and used as objective markers.

**Results:**

Irrespective of arm movement condition, children showed an increase in fear of falling and young adults a decrease in postural sway frequency when standing above ground than on ground level.

**Conclusion:**

The findings indicate that children are emotionally reactive but possibly not able to translate that into meaningful motor adaptation. Conversely, young adults react motorically but do not necessarily transfer that into an emotional response.

## 1 Introduction

Numerous studies (Adkin and Carpenter, 2018; Carpenter et al., 2001; Hill et al., 2023, 2024; Huffman et al., 2009; Laufer et al., 2006) have examined the influence of postural threat during static balance tasks, consistently demonstrating a “stiffening” response characterized by decreased center of pressure (COP) amplitude and increased COP frequency when standing at height (threat) compared to ground-level (no threat). In addition to these motor adaptations, a substantial body of evidence (Adkin and Carpenter, 2018; Ellmers and Young, 2018; Hill et al., 2023, 2024) have demonstrated that exposure to height-related postural threat elicits marked alterations in emotional state outcomes. Specifically, when individuals shift from standing at ground level to elevated positions, they consistently report increased fear of falling, a heightened perception of postural instability—reflected in reduced perceived safety—and an amplified engagement of conscious balance control processes (Adkin and Carpenter, 2018; Ellmers and Young, 2018; Hill et al., 2023, 2024). Together, these motor and psychological adaptations indicate that standing at elevated height challenges static postural control, triggering compensatory mechanisms in balance and emotional state outcomes.

While these motor and psychological adaptations have been extensively studied in young adults (Carpenter et al., 2001; Hill et al., 2023; Huffman et al., 2009; Laufer et al., 2006; Sturnieks et al., 2016), the effects on children have hardly been investigated to date. Importantly, assessing emotional state—such as fear of falling—is crucial in postural control research, as emotional responses can directly influence with motor performance. For example, heightened fear may shift attentional focus, increase postural stiffness, or promote maladaptive control strategies that compromise stability (Adkin and Carpenter, 2018; Hill et al., 2023; Huffman et al., 2009). Therefore, examining both emotional and motor responses offers a more comprehensive understanding of how individuals cope with postural threat. In addition, the two existing studies (Hill et al., 2024; Omorczyk et al., 2021) reported postural adaptations that differed from those reported in young adults. Specifically, children demonstrated opposite patterns of changes, i.e., increased COP amplitude and decreased COP frequency when standing at height compared to floor. These diverging results could be explained by the notion that children’s sensorimotor and emotional regulation systems are still maturing (De Sonneville et al., 2002; Hirabayashi and Iwasaki, 1995; Orendorz-Frączkowska and Kubacka, 2019; Perlman and Pelphrey, 2011; Shumway-Cook and Woollacott, 1985; Sinno et al., 2021), which may compromise their ability to effectively adapt to postural threats. Consequently, further research is needed to clarify how height-induced postural threat affects postural control and emotional state outcomes in pediatric populations and whether there is a general trend in the responses.

In addition to developmental differences in postural control, the ability to use the arms for compensatory movements plays an important role in maintaining balance. Several studies (Borgmann et al., 2025; Hébert-Losier, 2017; Hill et al., 2019, 2023; Objero et al., 2019) have demonstrated that restricting arm movements compared to free arm conditions increases COP amplitude, fear of falling, and perceived instability during static balance tasks. This is particularly relevant from a practical perspective, as arm movements are frequently constrained during everyday activities—for example, when individuals carry objects, use assistive devices, or stabilize items while standing or walking. These real-world constraints highlight the importance of understanding how restricted upper limb use impacts postural control mechanisms (Roostaei et al., 2022). It is plausible to attribute this phenomenon to the restriction of arm movements, which limits the redistribution of body mass, thereby reducing the moment of inertia (Roos et al., 2008) and decreasing the stability of the postural control system (Hill et al., 2019). Furthermore, this reflects a diminished ability to generate compensatory torques or counterbalancing movements (Marigold et al., 2003). If transferring these considerations to height-related impairments of postural control, it seems plausible to assume that these would be more pronounced under test conditions with restricted than with free arm movements. Hill and colleagues (Hill et al., 2023) provided evidence for compensatory mechanisms, whereby the availability of free arm movements at height resulted in attenuated CoP amplitude, diminished fear of falling, and lower perceived instability (reflecting greater perceived safety) in young adults, as compared to conditions under which arm movements were constrained. Notably, while the compensatory effects of free arm movements under height-induced threat have been analyzed in young adults (Hill et al., 2023), it remains unclear whether similar benefits will be detected in children, whose motor strategies may differ due to growth, maturation, and development (Malina et al., 2004).

Therefore, the present study compared the effects of free versus restricted arm movements on subjective (emotional state) and objective (postural control) indicators related to standing on ground (no threat) and at height (threat) between children and young adults. Based on prior findings (Carpenter et al., 2001; Hill et al., 2019, 2023, 2024; Huffman et al., 2009), we hypothesized that (i) height-induced postural threat would elicit detrimental effects on both static balance and emotional state outcomes, (ii) these effects would be more pronounced under restricted compared to free arm movement conditions, and (iii) these changes would be greater in children compared to young adults.

## 2 Materials and methods

### 2.1 Participants and sample size estimation

The estimation of the required sample size for the repeated measures analysis of variance (ANOVA) was conducted based on previous studies that examined the effects of height-induced postural threat and/or arm movements (Hill et al., 2023, 2024) on static postural control. G*Power software version 3.1.9.7 (Faul et al., 2007) was used and revealed that a minimum of 38 participants (*n* = 19 per age group) would be required to identify statistically significant postural threat by arm movement interactions (input parameters: effect size [*f*] = 0.25, significance level [α] = 0.05, power [1-β] = 0.80, number of groups = 2, number of measurements = 4, correlation among repeated measures *r* = 0.20). A total of 49 participants volunteered in this study (Table 1). Twenty-six children (9.8 ± 0.6 years) were recruited from a primary school and 23 young adults (24.7 ± 4.0 years) were enrolled from the student population of the host institution. All participants were free of musculoskeletal dysfunction, neurological impairment, or orthopedic disorder. Prior to participation, written informed consent was obtained from all participants and the parents of participating children. The study protocol was reviewed and approved by the Human Ethics Committee of the Faculty of Educational Sciences at the University of Duisburg-Essen (approval number: EA-PSY9/24/25032024).

**TABLE 1:** Characteristics of the participants by age group.

Characteristic	Children	Young adults
Sample size (*n*)	26	23
Gender (females; *n*)	13	11
Age (years)	9.8 ± 0.6	24.7 ± 4.0
Body height (cm)	141.6 ± 7.6	177.3 ± 10.1
Body mass (kg)	37.4 ± 10.1	75.6 ± 12.0

### 2.2 Experimental procedures

**FIGURE 1:**
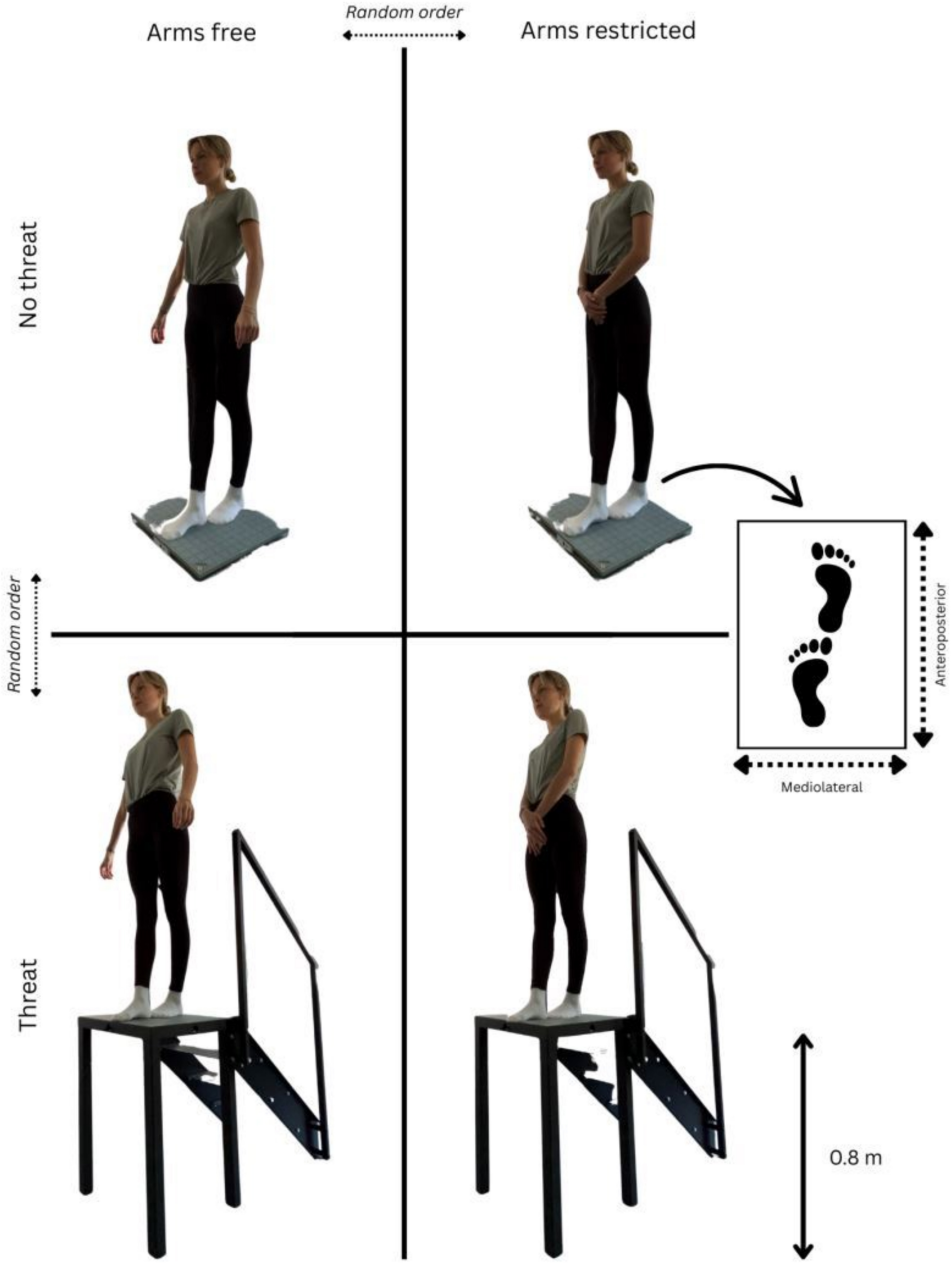
Schematic diagram of the postural threat (no threat vs. threat) and arm movement (free vs. restricted) conditions. The participants completed all trials in a tandem stance position. The individual that is shown has given written informed consent to publish these case details.

In line with one of our previous studies (Hill et al., 2023), participants completed static balance tasks in a tandem stance (right foot in front of the left foot) under varying postural threat conditions (Figure 1). The assessment was conducted under two different postural threat conditions while wearing no safety harness: (i) standing at ground level on a force platform (AMTI, AccuGait, Watertown, MA; dimensions 50 × 50 × 5 cm)–“no threat” condition and (ii) standing 80 cm above ground level on the elevated force platform—“threat” condition. Access to the elevated force platform was ensured via a staircase and was not equipped with any safety handrails, side rings, or barriers. This open-edge design was chosen to enhance the ecological validity of the postural threat and avoid artificially reducing perceived height-related risk. Each participant performed one 30-s practice trial followed by one 60-s data-collection trial per threat condition with free (i.e., arms moved freely) and restricted (i.e., hands clasped in front of the body at waist level) arm movements. All trials were performed without shoes to eliminate variability due to differences in footwear. The order of the four conditions was randomized, both in terms of the postural threat and arm movement conditions. Throughout each trial, participants were instructed to stand as still as possible and to maintain visual fixation on a black marker located 3 m in front of them at eye level. The design of the force platform and the instruction to position themselves in the center of the platform ensured that participants were approximately 15–20 cm from each side, minimizing the risk of lateral falls while maintaining the risk of perception in the anteroposterior (AP) direction.

### 2.3 Assessment of emotional state outcomes

Participants rated their confidence in maintaining balance and avoiding a fall during the static balance task using a visual analog scale (VAS) ranging from 0 (“not confident at all”) to 10 (“completely confident”), immediately before each trial (Lambrich et al., 2025). Following each trial, participants assessed their fear of falling experienced during the respective task using a VAS, where 0 correspond to feeling “not fearful at all” and 10 “completely fearful” (Lambrich et al., 2025). Subsequently, participants scored their perceived instability during the trial on another VAS ranging from 0 to 10 (0 = being “completely safe” to 10 = “so unsafe that I would fall”) (Ellmers et al., 2021; Huffman et al., 2009). Additionally, participants completed a 4-item questionnaire (i.e., a shortened version of the Movement Specific Reinvestment Scale; Masters et al., 2005) comprising four items designed to assess conscious balance processing: (1) “I always try to think about my balance when I perform this task”; (2) “I am aware of how my mind and body are functioning when performing this task”; (3) “I am aware of how I look when performing this task”; and (4) “I am concerned about my movement style when performing this task”. Each item was rated on a 6-point Likert scale ranging from 1 (“strongly disagree”) to 6 (“strongly agree”) (Ellmers and Young, 2018). To conduct the subsequent analysis, the total score (ranging from 4 to 24) was calculated, with higher scores indicating greater conscious processing of balance. VAS have been shown to be valid and reliable tools for assessing emotional responses such as fear of falling, perceived instability, and balance confidence in the context of postural control and height-induced threat (Adkin and Carpenter, 2018; Cleworth and Carpenter, 2016).

### 2.4 Assessment of static balance outcomes

During the tandem stance, ground reaction force data were recorded at a sampling rate of 1,000 Hz using an AMTI AccuSway optimized force platform (Watertown, MA, United States) and subsequently low-pass filtered offline using a second-order bidirectional Butterworth filter with a cut-off frequency of 5 Hz. The amplitude (mm) and frequency (Hz) of the COP displacement were quantified by calculating the root mean square (RMS) and mean power frequency (MPF) (Zaback et al., 2019) both the anteroposterior (AP) and the mediolateral (ML) directions. A decreased COP amplitude combined with an increased COP frequency when standing at height (threat) compared to ground-level (no threat) is indicative of a postural “stiffening” response (Adkin and Carpenter, 2018). Further, the mean COP velocity (mm/s) was calculated, whereas a decrease in the COP velocity represents an increase in the ability to maintain an upright stance (Le Clair and Riach, 1996; Palmieri et al., 2002).

### 2.5 Statistical analyses

The data were analyzed using JASP version 0.19.2. Assumptions of normality (Shapiro–Wilk test) and homogeneity of variance/sphericity (Mauchly test) were checked and met prior performing parametric analyses. Subsequently, a series of mixed model analyses of variance (ANOVAs) were conducted to examine the between-subject effect of age group (2 levels: children vs. young adults) and the within-subject effects of postural threat (2 levels: no threat vs. threat) and arm condition (2 levels: free vs. restricted). In cases where significant postural threat × arm movement condition interactions were identified; *post hoc* comparisons were performed using Bonferroni-adjusted alpha levels to localize specific pairwise differences. Effect sizes for the ANOVAs were reported as partial eta squared (η_*p*_^2^) and classified as small (0.02 ≤ η*_*p*_*^2^ ≤ 0.12), medium (0.13 ≤ η*_*p*_*^2^ ≤ 0.25), or large (η*_*p*_*^2^ ≥ 0.26). For pairwise comparisons, Cohen’s *d* was calculated (Cohen, 1988) and categorized as trivial (0 ≤ *d* ≤ 0.19), small (0.20 ≤ *d* ≤ 0.49), moderate (0.50 ≤ *d* ≤ 0.79), or large (*d* ≥ 0.80). The significance threshold (alpha level) for all statistical tests was set *a priori* at *p* < 0.05.

## 3 Results

Table 2 displays the group mean values ± standard deviations during no threat and threat conditions when standing with free compared to restricted arm movements and Table 3 presents the ANOVA outputs for all assessed.

**TABLE 2:** Group mean values ± standard deviations for all assessed variables by age group (children vs. young adults), threat conditions (no threat vs. threat), and arm movement strategies (free vs. restricted).

Parameter	Arm strategy	Children (*n* = 26)		Young adults (*n* = 23)	
		No threat	Threat	No threat	Threat
*Emotional state*					
Balance confidence (0–10)	Free	7.9 ± 2.1	6.8 ± 2.6	9.0 ± 1.4	8.3 ± 1.9
	Restricted	7.7 ± 2.1	6.5 ± 2.7	9.0 ± 1.4	7.6 ± 2.2
Fear of falling (0–10)	Free	2.2 ± 2.2	4.6 ± 2.9	0.9 ± 1.4	1.7 ± 2.0
	Restricted	2.0 ± 2.2	4.6 ± 2.9	1.0 ± 1.6	2.1 ± 2.2
Perceived instability (0–10)	Free	3.4 ± 2.3	4.8 ± 2.8	1.7 ± 1.6	2.0 ± 1.7
	Restricted	3.7 ± 2.3	4.3 ± 2.7	2.4 ± 2.2	1.6 ± 1.7
Conscious processing (4–24)	Free	14.6 ± 4.3	15.3 ± 5.0	14.6 ± 3.0	14.1 ± 3.3
	Restricted	14.7 ± 4.4	15.0 ± 4.7	14.6 ± 3.7	14.9 ± 2.8
*Static balance*					
AP COP RMS (mm)	Free	8.62 ± 3.93	8.89 ± 4.43	9.32 ± 5.14	6.79 ± 4.20
	Restricted	8.02 ± 3.42	8.17 ± 4.01	6.90 ± 2.67	6.89 ± 3.24
ML COP RMS (mm)	Free	6.70 ± 1.75	5.86 ± 1.51	5.65 ± 1.44	5.60 ± 1.45
	Restricted	5.80 ± 1.43	5.63 ± 1.17	5.18 ± 1.00	5.69 ± 1.91
AP COP MPF (Hz)	Free	0.21 ± 0.09	0.21 ± 0.09	0.29 ± 0.25	0.26 ± 0.15
	Restricted	0.20 ± 0.10	0.20 ± 0.08	0.34 ± 0.29	0.32 ± 0.16
ML COP MPF (Hz)	Free	0.26 ± 0.07	0.30 ± 0.07	0.40 ± 0.13	0.44 ± 0.15
	Restricted	0.27 ± 0.07	0.40 ± 0.14	0.31 ± 0.07	0.43 ± 0.16
AP COP vel (mm/s)	Free	10.80 ± 2.49	11.01 ± 2.41	16.35 ± 7.38	17.17 ± 5.68
	Restricted	9.74 ± 2.40	10.76 ± 1.65	14.72 ± 5.03	17.15 ± 6.06
ML COP vel (mm/s)	Free	10.80 ± 6.07	10.74 ± 4.83	15.83 ± 15.97	14.15 ± 5.50
	Restricted	9.38 ± 3.61	11.83 ± 5.40	9.91 ± 3.68	13.51 ± 4.59

AP, anteroposterior; COP, center of pressure; ML, mediolateral; MPF, mean power frequency; RMS, root mean square; vel, velocity.

**TABLE 3:** Main and interaction effects of the repeated measures ANOVA for all assessed variables.

Parameter	Group (children vs. young adults)	Threat (no threat vs. threat)	Arm (free vs. restricted)	Threat × Arm	Group × Threat	Group × Arm	Group × Threat × Arm
***Emotional state***							
Balance confidence (0–10)	***F* = 5.822** ***p* (*η*_***p***_^**2**^) = 0.020 (0.11)**	***F* = 29.048** ***p* (*η*_***p***_^**2**^) < 0.001 (0.38)**	***F* = 4.502** ***p* (*η*_***p***_^**2**^) = 0.039 (0.09)**	*F* = 2.499 *p* (*η*_p_^2^) = 0.121 (0.05)	*F* = 0.073 *p* (*η*_p_^2^) = 0.788 (0.00)	*F* = 0.154 *p* (*η*_p_^2^) = 0.697 (0.00)	*F* = 1.127 *p* (*η*_p_^2^) = 0.294 (0.02)
Fear of falling (0–10)	***F* = 13.095** ***p* (*η*_***p***_^**2**^) < 0.001 (0.22)**	***F* = 27.968** ***p* (*η*_***p***_^**2**^) < 0.001 (0.37)**	*F* = 0.005 *p* (*η*_p_^2^) = 0.942 (0.00)	*F* = 0.041 *p* (*η*_p_^2^) = 0.840 (0.00)	***F* = 4.604** ***p* (*η*_***p***_^**2**^) = 0.037 (0.09)**	*F* = 2.136 *p* (*η*_p_^2^) = 0.150 (0.04)	*F* = 0.499 *p* (*η*_p_^2^) = 0.483 (0.01)
Perceived instability (0–10)	***F* = 12.978** ***p* (*η*_***p***_^**2**^) < 0.001 (0.22)**	***F* = 14.969** ***p* (*η*_***p***_^**2**^) < 0.001 (0.24)**	*F* = 0.000 *p* (*η*_p_^2^) = 0.981 (0.00)	*F* = 0.190 *p* (*η*_p_^2^) = 0.665 (0.00)	*F* = 0.045 *p* (*η*_p_^2^) = 0.833 (0.00)	*F* = 0.221 *p* (*η*_p_^2^) = 0.640 (0.00)	*F* = 2.033 *p* (*η*_p_^2^) = 0.161 (0.04)
Conscious balance processing (4–24)	*F* = 0.101 *p* (*η*_p_^2^) = 0.753 (0.00)	*F* = 1.871 *p* (*η*_p_^2^) = 0.178 (0.04)	*F* = 0.256 *p* (*η*_p_^2^) = 0.615 (0.00)	*F* = 0.182 *p* (*η*_p_^2^) = 0.672 (0.00)	*F* = 0.037 *p* (*η*_p_^2^) = 0.848 (0.00)	*F* = 0.012 *p* (*η*_p_^2^) = 0.914 (0.00)	*F* = 1.524 *p* (*η*_p_^2^) = 0.223 (0.03)
***Static balance***							
AP COP RMS (mm)	*F* = 1.120 *p* (*η*_p_^2^) = 0.295 (0.02)	*F* = 1.137 *p* (*η*_p_^2^) = 0.292 (0.02)	***F* = 6.993** ***p* (*η*_***p***_^**2**^) = 0.011 (0.13**)	*F* = 2.636 *p* (*η*_p_^2^) = 0.111 (0.05)	*F* = 2.305 *p* (*η*_p_^2^) = 0.136 (0.05)	*F* = 0.649 *p* (*η*_p_^2^) = 0.409 (0.02)	*F* = 3.197 *p* (*η*_p_^2^) = 0.080 (0.06)
ML COP RMS (mm)	*F* = 2.065 *p* (*η*_p_^2^) = 0.157 (0.04)	*F* = 0.534 *p* (*η*_p_^2^) = 0.469 (0.01)	***F* = 6.884** ***p* (*η*_***p***_^**2**^) = 0.012 (0.13)**	***F* = 4.866** ***p* (*η*_***p***_^**2**^) = 0.032 (0.09)**	*F* = 3.915 *p* (*η*_p_^2^) = 0.054 (0.08)	*F* = 1.688 *p* (*η*_p_^2^) = 0.200 (0.04)	*F* = 0.037 *p* (*η*_p_^2^) = 0.848 (0.00)
AP COP MPF (Hz)	***F* = 7.545** ***p* (*η*_***p***_^**2**^) = 0.009 (0.14)**	***F* = 6.519** ***p* (*η*_***p***_^**2**^) = 0.014 (0.12)**	*F* = 0.699 *p* (*η*_p_^2^) = 0.407 (0.02)	*F* = 0.054 *p* (*η*_p_^2^) = 0.818 (0.00)	***F* = 5.582** ***p* (*η*_***p***_^**2**^) = 0.022 (0.11)**	*F* = 0.101 *p* (*η*_p_^2^) = 0.752 (0.00)	*F* = 0.000 *p* (*η*_p_^2^) = 0.989 (0.00)
ML COP MPF (Hz)	***F* = 30.670** ***p* (*η*_***p***_^**2**^) < 0.001 (0.39)**	***F* = 7.812** ***p* (*η*_***p***_^**2**^) = 0.007 (0.143)**	*F* = 0.043 *p* (*η*_p_^2^) = 0.837 (0.00)	*F* = 0.025 *p* (*η*_p_^2^) = 0.874 (0.00)	*F* = 0.021 *p* (*η*_p_^2^) = 0.887 (0.00)	*F* = 0.436 *p* (*η*_p_^2^) = 0.512 (0.01)	*F* = 0.163 *p* (*η*_p_^2^) = 0.688 (0.00)
AP COP vel (mm/s)	***F* = 4.827** ***p* (*η*_***p***_^**2**^) = 0.033 (0.93)**	*F* = 0.020 *p* (*η*_p_^2^) = 0.889 (0.00)	***F* = 5.303** ***p* (*η*_***p***_^**2**^) = 0.026 (0.10)**	*F* = 3.043 *p* (*η*_p_^2^) = 0.088 (0.06)	*F* = 0.019 *p* (*η*_p_^2^) = 0.891 (0.00)	*F* = 0.609 *p* (*η*_p_^2^) = 0.439 (0.01)	*F* = 1.424 *p* (*η*_p_^2^) = 0.239 (0.03)
ML COP vel (mm/s)	***F* = 24.767** ***p* (*η*_***p***_^**2**^) < 0.001 (0.34)**	***F* = 8.588** ***p* (*η*_***p***_^**2**^) = 0.005 (0.15)**	***F* = 7.306** ***p* (*η*_***p***_^**2**^) = 0.010 (0.14)**	***F* = 4.427** ***p* (*η*_***p***_^**2**^) = 0.041 (0.09)**	*F* = 1.752 *p* (*η*_p_^2^) = 0.192 (0.04)	*F* = 0.096 *p* (*η*_p_^2^) = 0.758 (0.00)	*F* = 0.484 *p* (*η*_p_^2^) = 0.490 (0.01)

0.02 ≤ *η*_p_^2^ ≤ 0.12 indicates small, 0.13 ≤ *η*_p_^2^ ≤ 0.25 indicates medium, and *η*_p_^2^ ≥ 0.26 indicates large effects. Bold values indicate statistically significant differences (*p* < 0.05). AP, anteroposterior; COP, center of pressure; ML, mediolateral; MPF, mean power frequency; RMS, root mean square; vel, velocity.

### 3.1 Emotional state outcomes

#### 3.1.1 Balance confidence

There were significant main effects for group (*p* = 0.020), threat (*p* < 0.001), and arm (*p* = 0.039), with participants nearly exclusively reporting lower balance confidence during the threat and restricted arm movement conditions. The interaction effects between these factors were not statistically significant.

#### 3.1.2 Fear of falling

The analysis detected significant main effects for group (*p* < 0.001) and threat (*p* < 0.001), with an interaction (*p* = 0.037, η*_*p*_*^2^ = 0.09) between both factors. *Post-hoc* tests revealed a significant increase in fear of falling during standing at height (irrespective of arm movement condition) in children (*t* = −5.425, *p* < 0.001, *d* = 1.04) but not in young adults (*t* = −2.157, *p* = 0.088, *d* = 0.44).

#### 3.1.3 Perceived instability

There were significant main effects for group (*p* < 0.001) and threat (*p* < 0.001), with no interaction between these factors. The participants reported almost exclusively greater perceived instability during the threat and restricted arm movement conditions.

#### 3.1.4 Conscious balance processing

There was neither a significant main effect nor a significant interaction effect.

### 3.2 Static balance outcomes

**FIGURE 2:**
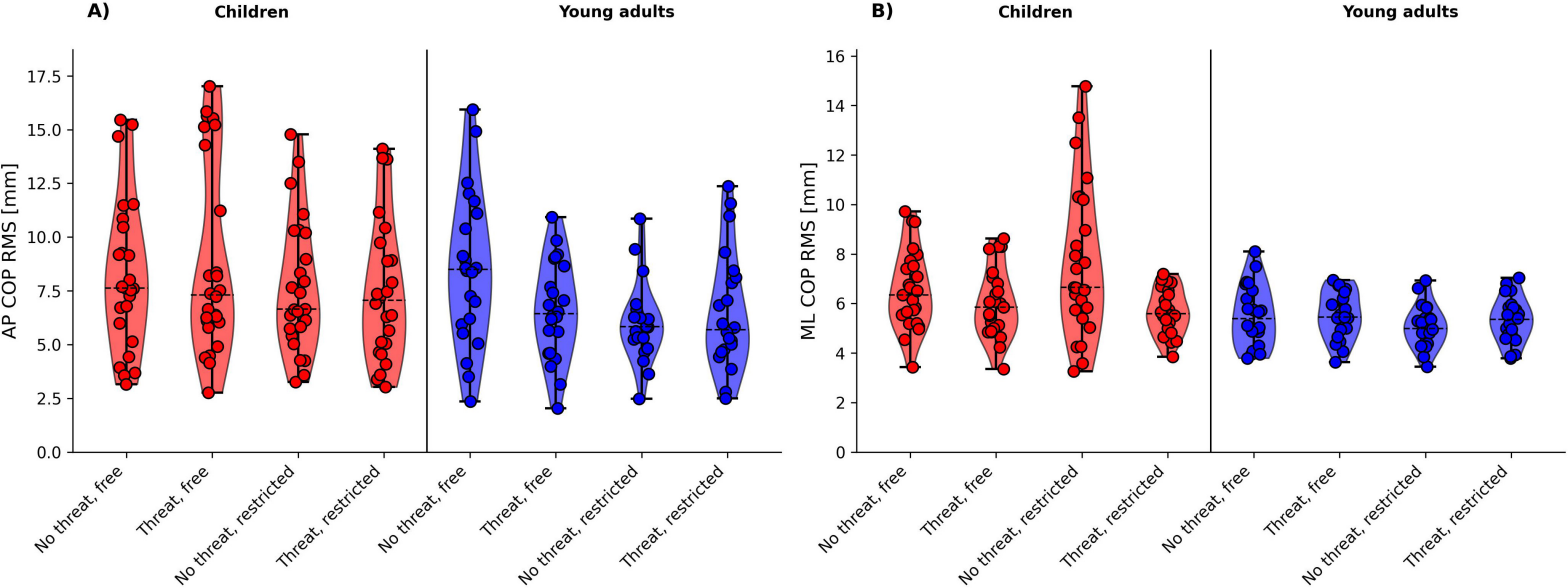
Violin plots of the COP amplitude for age group (children vs. young adults) and threat (no threat vs. threat) by arm movement (free vs. restricted) for **(A)** AP COP RMS and **(B)** ML COP RMS. The dashed line indicates the median.

**FIGURE 3:**
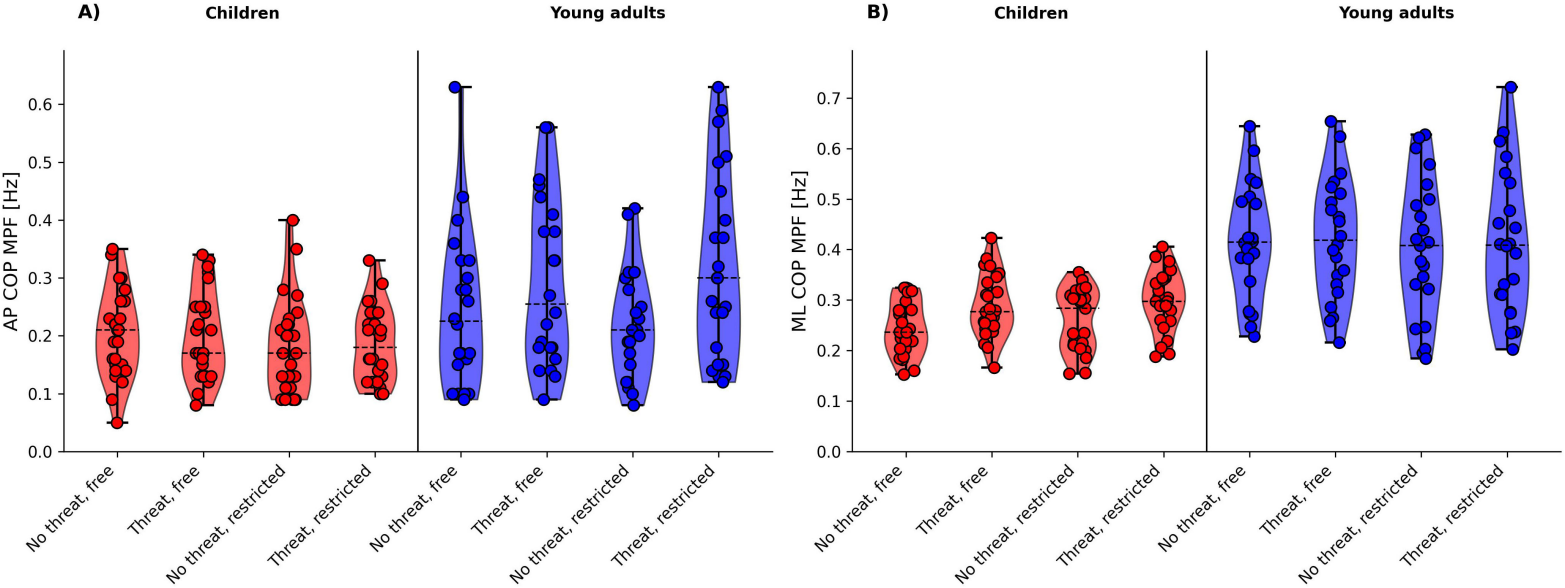
Violin plots of the COP frequency for age group (children vs. young adults) and threat (no threat vs. threat) by arm movement (free vs. restricted) for **(A)** AP COP MPF and **(B)** ML COP MPF. The dashed line indicates the median.

**FIGURE 4:**
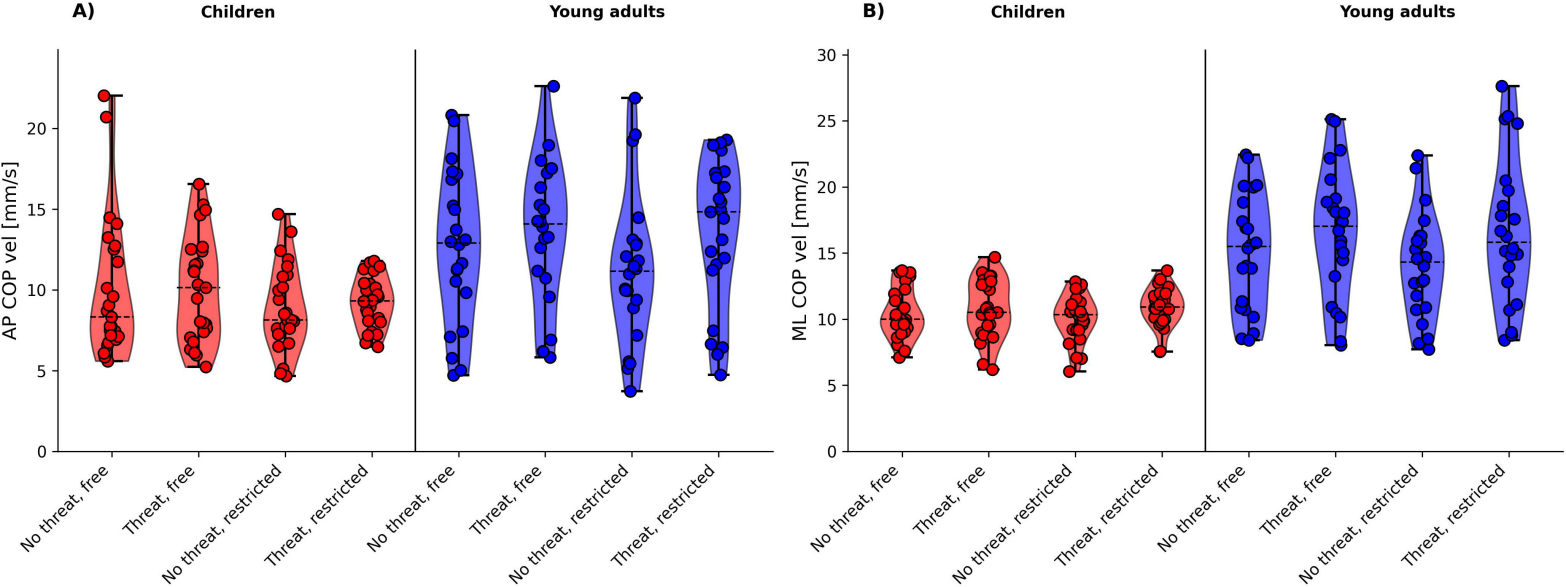
Violin plots of the COP velocity for age group (children vs. young adults) and threat (no threat vs. threat) by arm movement (free vs. restricted) for **(A)** AP COP vel and **(B)** ML COP vel. The dashed line indicates the median.

Figures 2A,B, 3A,B, 4A,B shows the violin plots of the COP amplitude, COP frequency, and COP velocity, respectively.

#### 3.2.1 COP amplitude (RMS)

The analysis revealed a significant main effect of arm with respect to AP COP RMS (*p* = 0.011) and ML COP RMS (*p* = 0.012), with participants showing a decrease in COP amplitude during the restricted arm movement conditions. Further, the threat × arm movement interaction was also significant for the ML COP RMS (*p* = 0.032). *Post-hoc* tests revealed a significant decrease in ML COP RMS during standing on ground (no threat) with free compared to restricted arm movements only (*t* = 2.965, *p* = 0.028, *d* = 0.46).

#### 3.2.2 COP frequency (MPF)

There was a significant main effect of group for both AP COP MPF (*p* = 0.009) and ML COP MPF (*p* < 0.001), with young adults showing larger values than children. Further, we observed a significant main effect of threat for both AP COP MPF (*p* = 0.014) and ML COP MPF (*p* = 0.007), with participants showing a decrease in AP COP MPF and an increase in ML COP MPF during the threat conditions. With respect to AP COP MPF, there was a significant group × threat interaction. *Post hoc* tests revealed a significant decrease in frequency of COP displacements during standing at height (irrespective of arm movement condition) in young adults (*t* = −3.374, *p* = 0.008, *d* = 0.36) but not in children (*t* = −0.139, *p* = 0.890, *d* = 0.01).

#### 3.2.3 COP velocity

The analysis yielded a significant main effect of group for both AP COP vel (*p* = 0.033) and ML COP vel (*p* < 0.001), with young adults showing larger values than children (Table 2). With respect to ML COP vel (*p* = 0.005), there was a significant main effect of threat, with participants showing an increase in velocity of COP displacements during the threat conditions. In addition, we detected a significant main effect of arm for both AP COP vel (*p* = 0.026) and ML COP vel (*p* = 0.010), with participants showing a decrease in COP velocity during the restricted arm movement conditions. Further, the threat × arm movement interaction was also significant for the ML COP vel (*p* = 0.041). *Post hoc* tests revealed a significant decrease in ML COP vel during standing on ground (no threat) with free compared to restricted arm movements only (*t* = 3.052, *p* = 0.015, *d* = 0.30).

## 4 Discussion

The present study aimed to test the hypotheses that (i) height-induced postural threat would detrimentally affect both static balance and emotional state outcomes, (ii) these effects would be more pronounced under restricted compared to free arm movement conditions, and (iii) these changes would be greater in children compared to young adults. Our findings offered partial support for these hypotheses, though several unexpected findings emerged that warrant closer examination.

More specifically, a significant interaction between group and threat for static balance performance was observed. Young adults showed a significant reduction in sway frequency under postural threat, whereas no significant changes were observed in children. The reduction in COP frequency (AP direction) in young adults, contrary to prior findings using the same tandem stance (Hill et al., 2023), suggests a potential shift in postural control strategy toward slower, more deliberate postural adjustments. In other words, this reflects a strategic adaptation aimed at enhancing postural stability through refined motor control, rather than relying on the rapid, high-frequency corrections typically associated with increased postural threat levels (Ellmers et al., 2022; Hill et al., 2024; Laufer et al., 2006; Sturnieks et al., 2016). One possible explanation is that young adults, when faced with postural threat, adopted a more cautious, conscious control strategy—slowing down postural sway as a means of maintaining stability—rather than resorting to co-contraction (Cleworth et al., 2016; Lelard et al., 2014; Zaback et al., 2019) or “stiffening” strategy (Adkin and Carpenter, 2018; Carpenter et al., 2001; Hill et al., 2023, 2024; Huffman et al., 2009; Laufer et al., 2006) as previously reported. However, the interpretation of threat-related reductions in COP frequency in the AP direction is speculative and partially not supported by our data, as no group-by-threat interaction was found with regard to COP velocity. It is possible that COP frequency decreased due to an increase of the power of the low-frequency content, without changing the amplitude of the high-frequency content, or vice versa. Therefore, it cannot be definitively said that the detected threat-related reductions in COP frequency in the AP direction reflect slower postural adjustments. An alternative explanation is that the threat manipulation did not elicit a meaningful emotional response in the young adult group, and thus the effect of threat may have been insufficient to increase COP frequency as is typically observed (Adkin and Carpenter, 2018; Carpenter et al., 2006; Cleworth and Carpenter, 2016). Previous work from the Carpenter group (Adkin and Carpenter, 2018; Carpenter et al., 2006; Cleworth and Carpenter, 2016) has shown that threat-related changes in high-frequency COP adjustments—and consequently MPF—are tightly coupled with the emotional responses to threat, more so than other COP outcomes. Accordingly, the finding that young adults exhibited a decrease rather than an increase in COP frequency in the AP direction may simply reflect the absence of an emotional response to the postural threat. Together, the lack of an emotional response to threat, combined with the fact that individuals have an extended AP base of support during the used tandem stance task could explain why COP frequency did not increase when exposed to height-induced postural threat.

In children, the absence of significant balance adjustments stands in contrast to previous work using the bipedal stance (Hill et al., 2024). The more challenging tandem stance employed here may have played a critical role. The tandem stance likely imposed a substantial postural challenge, potentially pushing children close to the limits of their available capacity of postural control and thereby constraining their ability to further modulate balance in response to the added threat. The high task demand may have effectively “masked” or constrained any potential threat-related adaptations in postural control that were observable under less demanding conditions. In less demanding tasks (e.g., bipedal stance), children have “room” to display maladaptive strategies such as increased amplitude and decreased frequency; here, however, maintaining balance alone may have taxed their available resources.

Regarding emotional state outcomes, a significant group-by-threat interaction was observed, indicating discrepancies between age groups. More precisely, children reported significantly greater increases in fear of falling under threat conditions, while young adults did not show significant emotional changes. This finding supports the notion that children are emotionally reactive to postural threat but may lack the neuromuscular capacity to translate this heightened emotional state into effective motor adaptation, particularly under high task demands such as those imposed by the challenging tandem stance. Conversely, young adults demonstrated motor adaptations to height-induced postural threat without concomitant increases in fear of falling, suggesting a dissociation between emotional and motor responses in this population.

Contrary to above-mentioned expectations, restricting arm movement did not amplify threat-related effects in either balance or emotional outcomes, nor did it interact with age group to modulate responses. This may reflect a habituation effect resulting from the relatively long stance duration employed in the current study (i.e., 60 s) compared to those used by Brown et al. (2006) (i.e., 15 s). In this regard, Zaback et al. (2019), Zaback et al. (2021) and Zaback et al. (2025) demonstrated that repeated exposure to the same height-induced postural threat led to substantial adaptations in emotional state and balance parameters.

The key strengths of the present study include the comparison of subjective (i.e., emotional state outcomes) and objective (i.e., balance outcomes) indicators related to static postural control. In addition, by using an identical study design (i.e., dual-group repeated-measures design) and task conditions (i.e., tandem stance at and 80 cm above ground level), we (i) replicated previous findings (Hill et al., 2023) and (ii) provided new insights into understanding how children compared to young adults behave under postural threat and arm restriction while standing. Despite these strengths, the present study has some limitations. First, we investigated only healthy children and young adults, which limits the generalizability of our findings to older, fall prone or neurologically impaired individuals (e.g., children with cerebral palsy). Second, while we assessed the effects of different arm movement strategies using questionnaires (i.e., VAS) and biomechanical assessment (i.e., instrumented force-plate), we did not include physiological measures (i.e., brain/muscle activity), which constrains our ability to draw conclusions about underlying mechanisms.

## 5 Conclusion

In conclusion, the present findings indicate that children exhibit pronounced emotional responses to postural threat but are limited in their ability to translate these responses into meaningful motor adaptations under high task demands (i.e., tandem stance). Conversely, young adults demonstrated motor adjustments without corresponding changes in emotional state. These preliminary findings highlight the complex, context-dependent interaction between emotional and motor responses to postural threat across developmental stages. Future studies employing more dynamic postural tasks (e.g., leg swing during unipedal stance) are required to further explore these aspects and to determine whether similar patterns persist under varying task demands. In addition to extending this work to more dynamic balance tasks, future research should also explore similar paradigms in clinical populations. For example, children with neuromotor impairments—such as cerebral palsy—often experience compromised postural control and are particularly vulnerable to falls, especially when upper extremity movements are restricted or when environmental threat levels are heighted. Examining how height-induced postural threat and arm movement constraints affect both emotional state and postural control outcomes in such populations could help to develop targeted, ecologically valid rehabilitation strategies (Roostaei et al., 2022).

## Data Availability

The raw data supporting the conclusions of this article will be made available by the authors, without undue reservation.
